# Evaluation of forest ecosystem resilience to drought considering lagged effects of drought

**DOI:** 10.1002/ece3.70281

**Published:** 2024-09-10

**Authors:** Qingfeng Xu, Ruyue Yu, Lili Guo

**Affiliations:** ^1^ Yellow River Engineering Consulting Co., Ltd. Zhengzhou China; ^2^ College of Land Science and Technology China Agricultural University Beijing China

**Keywords:** drought, forest ecosystem, lagged effects, recovery

## Abstract

Drought can cause significant disruption to forest ecosystems and may have long‐term impacts on the structure and function of ecosystems after the end of drought. This is the key to quantifying the ability of ecosystem to respond to disturbance events by comprehensively analyzing the impact of drought on vegetation, the lagged effect, and ecosystem resilience to drought. This article takes broad‐leaved forests and coniferous forests in multiple temperature zones of China as the object of study, using distributed lagged nonlinear model (DLNM) to construct a systematic method. Our results show that the main sensitive lagged time for coniferous forests and broad‐leaved forests is the first 3 months in various temperature zones, with the strongest lagged effect in the month when the drought incidents occur. Coping capacity represents ecosystems to remain stable during droughts, and we quantified the indicator by the ratio of the resistance (the difference between NDVI value before the drought and during the drought) to recovery (the difference between NDVI value after the drought and during the drought). When dealing with intensive drought events, the coping capacity of subtropical broad‐leaved forests (−0.67) and tropical broad‐leaved forests (−0.88) exhibit the strongest coping capacity (value tends to −1). Overall, vegetation growth in subtropical and tropical regions is less affected by drought compared to temperate and cold temperate zones. The research results help us understand the comprehensive impact of drought on vegetation and the strategies for different vegetation to cope with drought.

## Graphical abstract

This article develops a quantitative evaluation method that systematically analyzes the impact of drought on vegetation, the lagged effect of drought, and ecosystem resilience to drought. The inhibitory effect of intensive drought shows a gradient increase along the drought intensity in various temperature zones. The main sensitive lagged time for coniferous forests and broad‐leaved forests is the first 3 months in various temperature zones, with the strongest lagged effect in the month when the drought incidents occur. When dealing with intensive drought events, the coping capacity of subtropical broad‐leaved forests (−0.67) and tropical broad‐leaved forests (−0.88) exhibit the strongest coping capacity (value tends to −1).

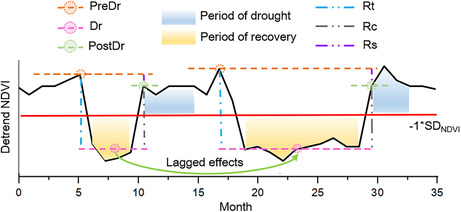

## 1 INTRODUCTION

Disturbances shape the structure, composition, and distribution of vegetation globally, and it may exert far‐reaching impacts on the function and stability of forest ecosystems (Ding et al., 2012; Keeley et al., 2019). Drought and associated biotic and abiotic disturbances have emerged as one of the most important driving factors (Anderegg et al., 2019; Turner, 2010). Drought can affect vegetation through a variety of different pathways, for example, by inhibiting photosynthesis and altering respiration. In addition, droughts can lead to a reduction in vegetation cover, mortality, and potential flips between carbon source and carbon pool (He et al., 2011; Li, Tong, et al., 2020). Different forest ecosystems are likely to vary in the magnitude and direction of their responses to drought due to differences in plant species in terms of turnover times and drought adaptation strategies (Schwalm et al., 2012; Zhang et al., 2021). It has been found to conclude that conifers should be assigned a lower drought resilience than broadleaved species in parts of the world based on data extracted from the global literature (Schwarz et al., 2020; Wu et al., 2014). However, it is not clear whether vegetation types follow similar or unique patterns of drought vulnerability in different climate zones. This is of great practical significance for drought impact assessment (Yao et al., 2022).

The intensity, duration, and recovery determine the impact of drought on vegetation (Ruppert et al., 2015; Xu et al., 2018). The Standardized Precipitation Evaporative Dispersion Index (SPEI) is an important indicator for assessing regional drought because it can identify changes in evapotranspiration and temperature (Dai, 2011; Ding et al., 2020). However, the effects of drought can persist after the end of a drought event (Frank et al., 2015). These post‐drought impacts are often referred to as the lagged effect and have been demonstrated for various aspects of ecosystem structure (Müller & Bahn, 2022; Vilonen et al., 2022; Xu et al., 2018). For example, *Quercus* sp. or *Fagus sylvatica* sometimes show growth minima only 1 year after the actual drought, which may lead to temporal misalignments of the identified drought (Galiano et al., 2011; Schwarz et al., 2020). It needs to be considered how to quantify the lagged effect of drought on vegetation (Müller & Bahn, 2022; Zhang et al., 2017).

Calculating ecosystem resistance and recovery in the event of drought is key to quantifying ecosystem response to disturbance (Gazol et al., 2017). The attempt to quantify ecosystem responses to disturbance has led to the development of a large number of indicators of resilience. In terms of forest ecosystems, satellite remote sensing data and ground observation data such as tree rings provide information on vegetation growth during drought periods (Li, Piao, et al., 2020; Li, Tong, et al., 2020; Lloret et al., 2011; Schwarz et al., 2020). Based on these data, assessing the response of forest ecosystems to drought includes the following components: resistance, recovery, resilience, and etc. Resistance is considered to be the reversal of the decline in ecological performance during disturbance (Chang et al., 2018). Recovery refers to the ability to recover relative to the damage experienced during the disturbance, and resilience refers to the ability to reach pre‐disturbance performance levels (He et al., 2018; Hodgson et al., 2015). A study on gymnosperms showed that their resilience to drought increased significantly with the duration of drought, but their resistance gradually decreased (Li, Piao, et al., 2020; Li, Tong, et al., 2020). However, further research is needed on the trade‐off between the resilience and resistance of various vegetation to drought in different temperature zones, and it is necessary to conduct research on a larger spatial scale (Yao et al., 2022; Zhang et al., 2021). On this basis, Schwarz et al. (2020) proposed a new analytical framework called “line of full resilience” which integrated the three most commonly used resilience indices and ranked of different tree species or treatments.

Considering that the effects of drought can last for months or even up to a year, the commonly used single‐month lagged or moving‐averaged lagged structure methods do not capture actual exposure and can lead to errors and bias in risk estimates (Fu et al., 2023). In order to accurately capture the lagged effects of drought, we introduced the distributed lag nonlinear model (DLNM). Due to its flexibility in assessing nonlinear exposure‐response relationships and lagged effects, DLNM has been widely used to study the effects of daily environmental factors (Chen et al., 2019; Yan et al., 2023; Yang et al., 2021). This article takes broad‐leaved and coniferous forests in multiple temperature zones of China as the object of study, introduces DLNM and framework named line of full resilience to quantitatively evaluate the lagged effect of drought, and further studies the resilience, recovery, and resistance changes of various forest ecosystems to different drought intensities.

## 2 DATA AND METHODS

### 2.1 Datasets

**FIGURE 1:**
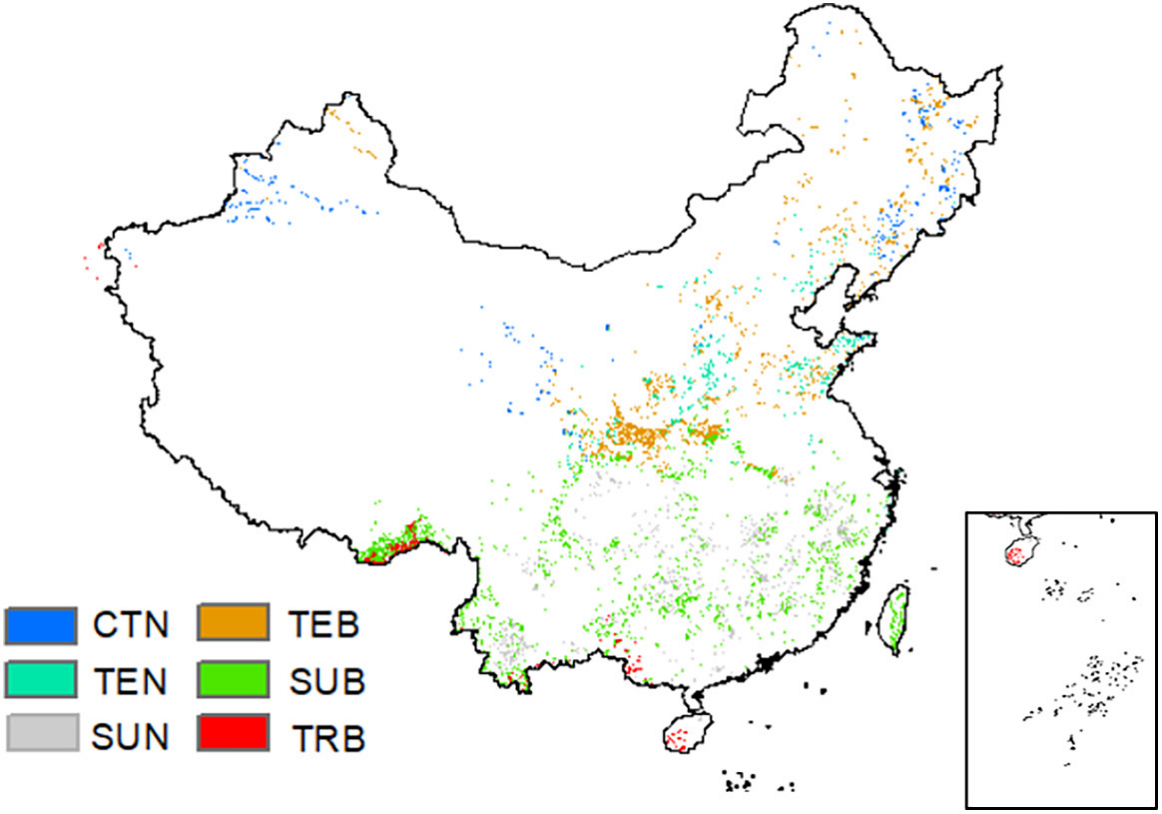
Spatial distribution data of vegetation types in China (CTN, cold temperate coniferous forest; SUB, subtropical broad‐leaved forest; SUN, subtropical coniferous forest; TEB, temperate deciduous; TEN, temperate coniferous forest; TRB, tropical broad‐leaved forest).

The dataset of spatial distribution of 1 million vegetation types in China was obtained from the Resource and Environment Data Center of the Chinese Academy of Sciences (CAS), which reflected the distribution of 11 vegetation type groups and 54 vegetation units in China, as well as the horizontal and vertical zonation patterns (https://www.resdc.cn/data.aspx?DATAID=122). In this study, we focused on forest ecosystems and selected typical vegetation, including coniferous forests located in cold temperate, temperate, and subtropical regions, and broad‐leaved forests were located in temperate, subtropical, and tropical regions (Figure 1), while the spatial resolution is 1 km.

In evaluating drought, we used the first high‐resolution monthly SPI/SPEI dataset developed by Zhang et al. (2023) in China from 1982 to 2015, which has been validated to perform well in identifying both long‐term and short‐term droughts. This dataset can be available for long‐term (over 24 months) at a monthly temporary resolution. Its advantage is that it can more accurately identify mild drought events, while the spatial resolution is 0.1°. This high spatiotemporal resolution SPI/SPEI dataset is helpful for refined drought monitoring research and is also suitable for sudden droughts. It also provides data support for studying the impact of drought on ecosystems, crops, hydrology, and other fields.

In evaluating vegetation growth, we chose to use NDVI data for analysis. The NDVI dataset is the latest release of the long sequence (1982–2015) normalized difference vegetation index product of NOAA Global Inventory Monitoring and Modeling System (GIMMS), version number 3 g.v1. The temporal resolution of the product is twice a month, while the spatial resolution is 1/12°. The temporal coverage is from July 1981 to December 2015. Due to its long‐time range, it has been widely used in global vegetation monitoring (Chu et al., 2019; Li, Piao, et al., 2020; Li, Tong, et al., 2020). We used resampling methods to process the data to match the resolution of vegetation types, NDVI, and SPEI data.

### 2.2 Methods

#### 2.2.1 Definition of drought events

**FIGURE 2:**
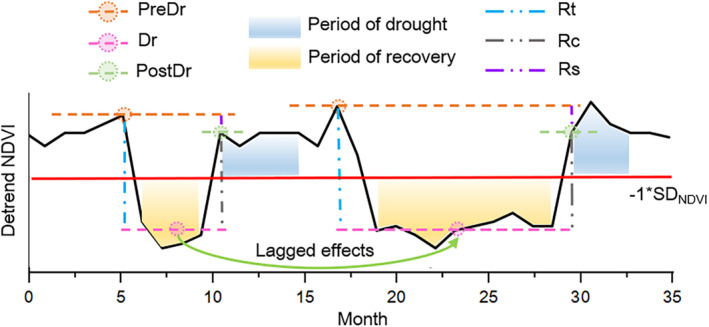
Curve diagram of the SPEI and detrended NDVI in a time series. Blue shadowing represents the occurrence of drought and NDVI anomalies, and yellow shadowing represents the period of recovery after drought. Each drought event has a corresponding calculation method to obtain resistance (Rt), recovery (Rc), and resilience (Rs) based on the de trending NDVI values of different periods, pre drought (PreDr), period of drought (Dr), and post drought (PostDr).

In order to avoid the impact of seasonal changes, we used the “detrend” function to remove the NDVI trends in MATLAB 2018. Vegetation growth anomalies were defined as a detrended NDVI below a standard deviation of −1 (−1 SD). Drought intensity (*T*, Equation 3) below −1 was defined as drought (Rhee & Im, 2017). Only when abnormal vegetation growth and drought occur simultaneously and last for at least 2 months or more will it be defined as the beginning of a drought event in order to avoid the occurrence of drought when the vegetation itself is abnormal (Schwarz et al., 2020; Yao et al., 2022). The restoration of normal vegetation growth for at least 2 months is defined as the end of the drought, and the duration of the period is considered the period of drought occurrence and recovery, respectively (Zhang et al., 2021) (Figure 2).

#### 2.2.2 Evaluation of the lagging effect

When evaluating the impact of the lagged effects of previous droughts on current drought events, based on experimental observations such as oak trees, we first assume that all drought events that occurred within the past 12 months may have had an impact on the current drought. It can be expressed by the Formula (1).

(1) $$\mathrm{lag}\left(t\right)=f\left(x_{t},x_{t-1},x_{t-2},\ldots x_{t-L}\right)$$

where lag (*t*) is the NDVI detrend value of vegetation at a certain drought time *t*, *x* means the lag effect of drought on the current NDVI, and the *L* value is taken as 12.

To further determine the lagged effect of drought, we quantified the lagged effect of drought using the DLNM model.

(2) $$\mathrm{Log}\left(\mu_{t}\right)=\alpha \left(t\right)+\mathrm{cb}\left(\text{SPEI},\mathrm{lag},\mathrm{df}\right)+\mathrm{ns}\left(\text{SPEI},\mathrm{df}\right)$$

where *μ*_*t*_ is the expected NDVI detrend value on month *t*. A crossbasis function (cb) was constructed using a natural cubic spline (NCS) function with a lag reaction dimension of 3 degrees of freedom (df). These nodes are placed at the same intervals on the logarithmic scale of the lag day to allow for more flexibility at the beginning of the distribution lag curve and are expected to have more changes (Gasparrinia et al., 2010). ns (SPEI, df) is a natural cubic spline applied to the 12 months moving average of SPEI, 3 df to control the mixed effects of drought; *α*(*t*) is the intercept (Fu et al., 2023). Based on the results obtained from the model, we can obtain the comprehensive impact of different SPEI on detrended NDVI, which may be positive or negative.

Therefore, we defined a new drought intensity *T* to more accurately represent the drought risks faced by vegetation according to the comprehensive impact allfit_*t*_. The new intensity *T* is aimed at emphasizing the lag effect of drought on vegetation.

(3) $$T=\sum_{\;t=t_{s}}^{t_{e}}{\text{SPEI}}_{t}\times {\text{allfit}}_{t}$$

Among them, *t*_s_ is the start time of drought, *t*_e_ is the end time of drought, SPEI_*t*_ is the SPEI value of *t* during the drought time, and allfit_*t*_ is the corresponding impact value of SPEI_*t*_.

#### 2.2.3 The frequency of drought events

The calculation of drought frequency is based on the determination of drought intensity *T*, NDVI values, and duration.

$$F=\sum_{\;t=t_{s}}^{t_{e}}f\left(t\right)$$

(4) $$f\left(t\right)=\left\{\begin{array}{c} 1,\text{if}\;T_{t}\leq -1\;\text{and}\;\mu_{t}<-1*\mathrm{SD}\;\text{and}\;D_{t}\geq 2\\ 0,\text{others} \end{array}\right.$$

where *t*_s_ is the start time of drought, *t*_e_ is the end time of drought, *T*_*t*_ is the drought intensity on month *t*, *μ*_*t*_ is the NDVI detrend value on month *t*, and $D_{t}$ is the duration months of drought.

#### 2.2.4 Capacity of different ecosystems to cope with drought events

In this study, we modified the calculation of resistance (Rt), recovery (Rc), and resilience (Rs) of vegetation to avoid the effect of positive and negative values as the NDVI data were detrended (Lloret et al., 2011).

(5) Rt=Dr−PreDr

(6) Rc=PostDr−Dr

(7) Rs=PostDr−PreDr

where Dr is the average of the detrended NDVI value during the drought, PreDr is the detrended NDVI value before the drought, and PostDr is the detrended NDVI value after the drought.

To further analyze the impact of large‐scale drought events on vegetation, we selected drought events with a duration of drought and a recovery time greater than or equal to 3 months. Based on the theory of line of full resilience, the resilience is set to 0 so that the vegetation is completely recovered after the occurrence of drought, which is the line of full resilience (Equation 7). The framework is a promising method for summarizing the response of trees to drought growth, which has been demonstrated in detail by Schwarz et al. (2020). The ratio of the Rc to Rt means ecosystem capacity to cope with drought. When the value is closer to the line of full resilience (value of −1), it means that the ability to cope with drought is stronger.

(8) Rc=−Rt

## 3 RESULTS

### 3.1 Evaluating lagged effects of drought

**FIGURE 3:**
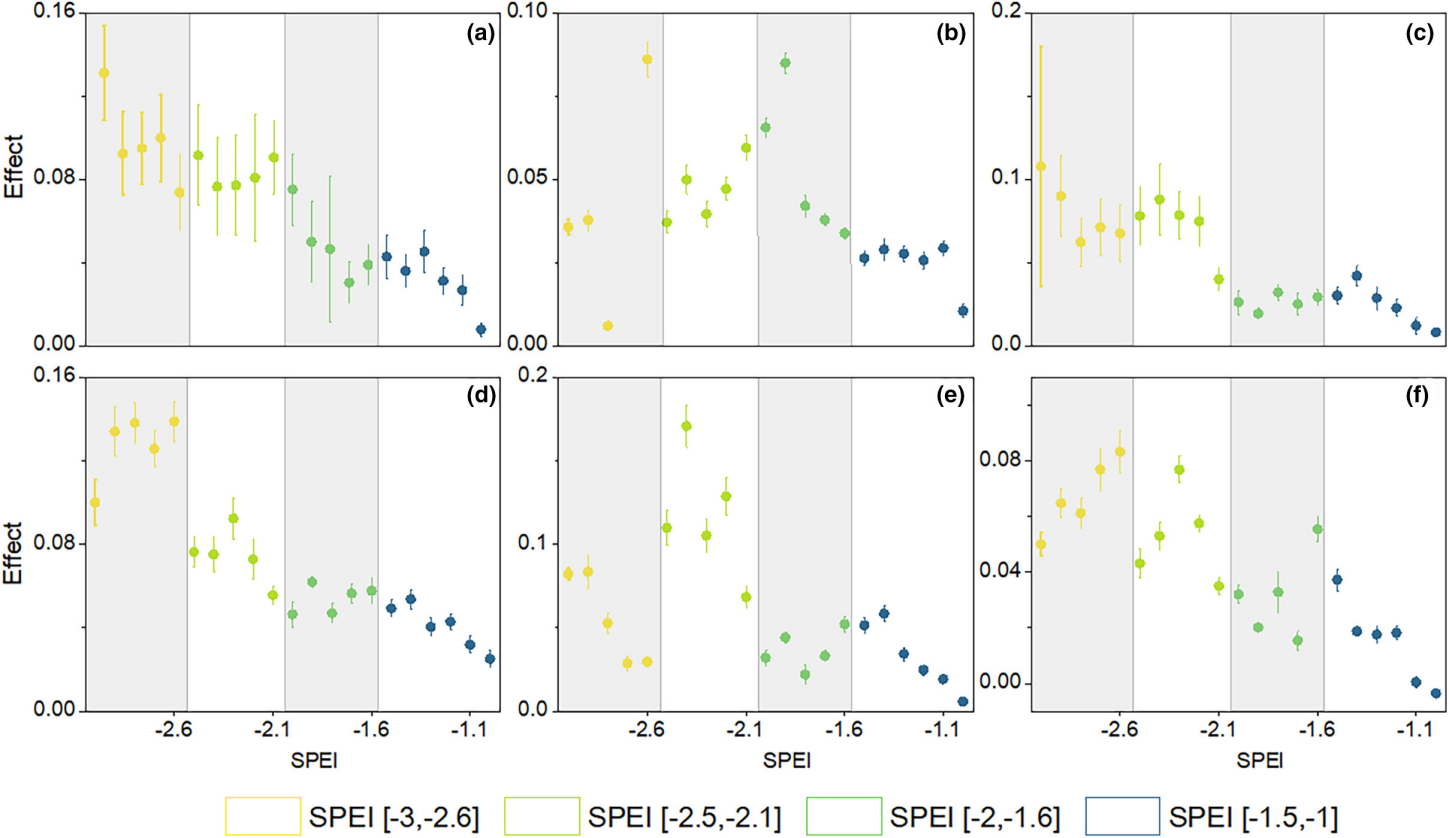
Percentage changes (with 95% CIs) of maximum growth limitation on vegetation under four drought intensities (SPEI range: [−3, −2.6], [−2.5, −2.1], [−2, −1.6], and [−1.5, −1]) using DLNM for different forest types.

Different drought intensities have varying growth limitations on various broad‐leaved and coniferous forests (SPEI < −1) (Figure 3). At higher drought intensities (−3 < SPEI < −2), it can be found that coniferous forests are relatively more growth‐limited. Similarly, as the drought intensity gradually decreased towards 1, the growth limitation of broadleaf and coniferous forests was gradually alleviated in all temperature zones.

**FIGURE 4:**
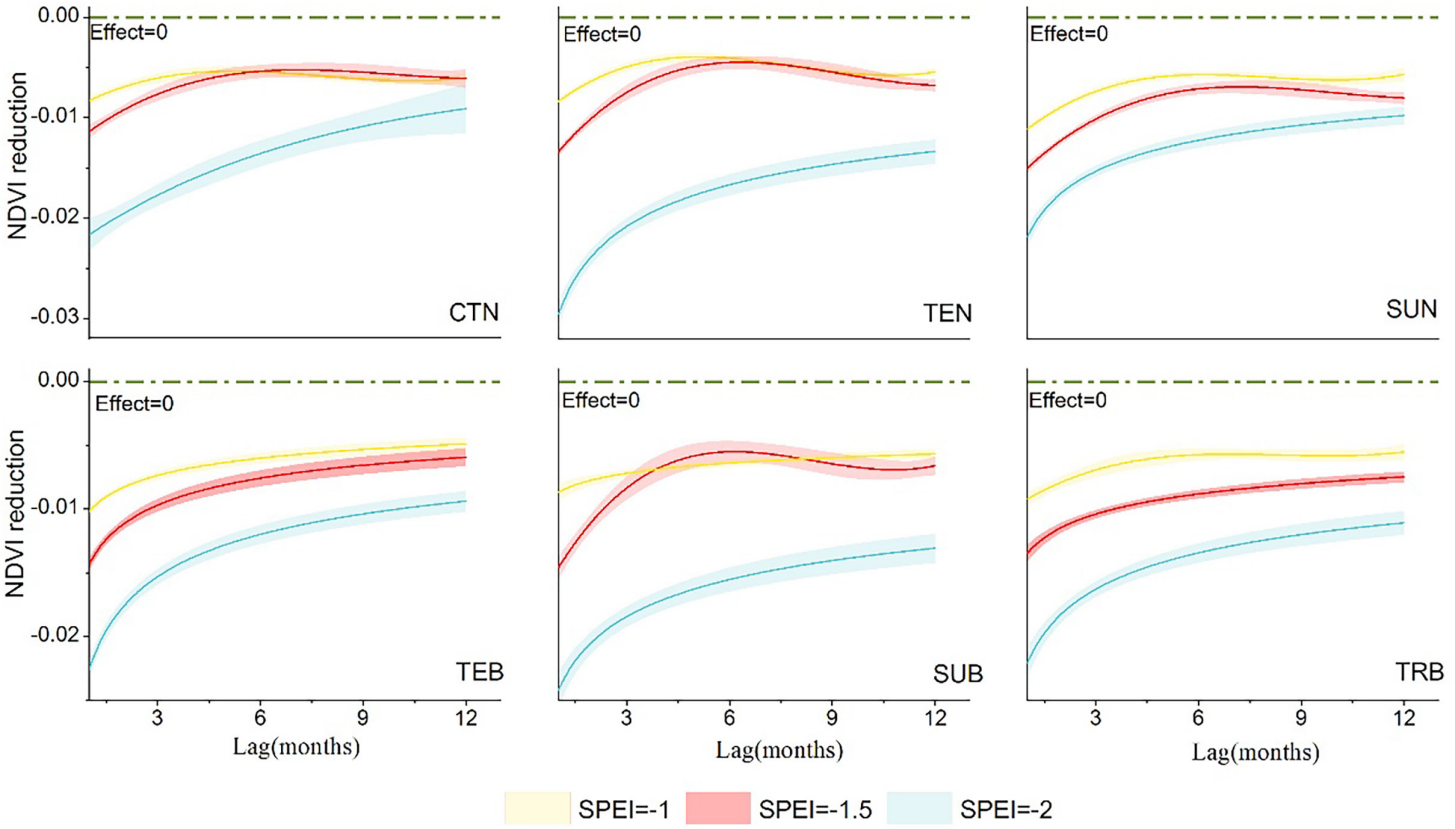
Lagged effect curve represents the relationship of NDVI reduction and lag time (month) for three drought intensities for different forest types. The blue, red, and yellow curve represents SPEI = −2, −1.5, −1, respectively.

Furthermore, we compared the lagged effects of drought within 12 months under the more frequent drought intensity gradient (SPEI = −2, −1.5, −1) (Figure 4). Overall, the maximum inhibition of plant growth for each drought intensity still occurred in the month of drought occurrence, and the cumulative effect of drought intensity at SPEI = −2 was significantly higher than that of SPEI = −1.5, −1 in the first 3 months. After 6 months, we can observe that the cumulative impact of the three drought intensities gradually decreases and tends towards around −0.01.

### 3.2 Drought frequency and characteristics of vegetation in different temperature zones

**FIGURE 5:**
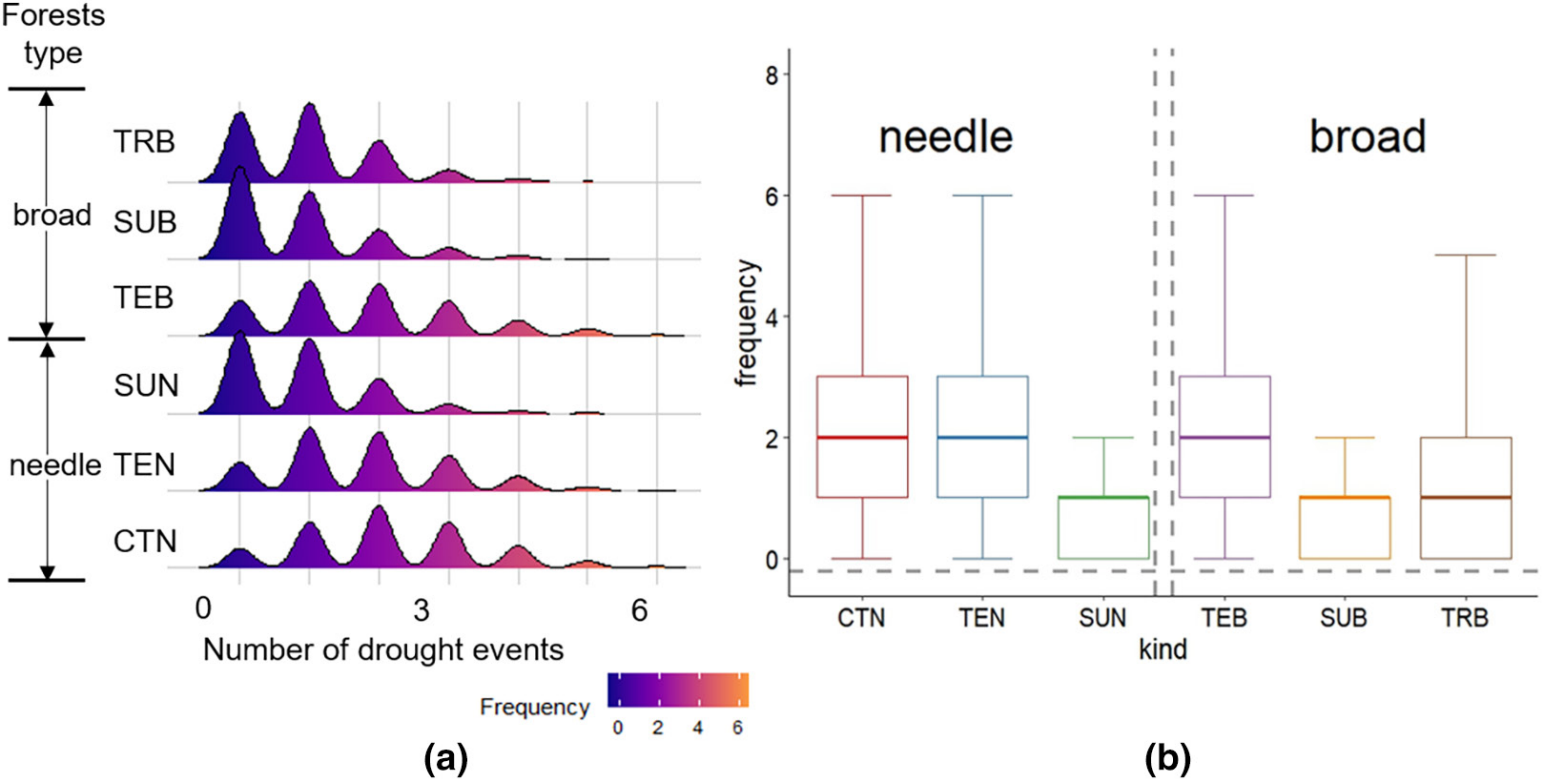
Frequent of drought for different forest types throughout the study period from 1982 to 2015.

Based on the definition of drought events, it shows the frequency of drought events in broad‐leaved and coniferous forests of different temperature zones between 1982 and 2015 (Figure 5). It can be found that vegetation drought events in subtropical and tropical regions occur mostly 0–1 time, while vegetation drought events in temperate and cold temperate zones mainly occur 1–3 times (Figure 5a). The final average frequency of drought occurrence is CTN (2.16), TEB (1.87), TEN (1.81), TRB (1.02), SUN (0.91), and SUB (0.86) (Figure 5b).

**FIGURE 6:**
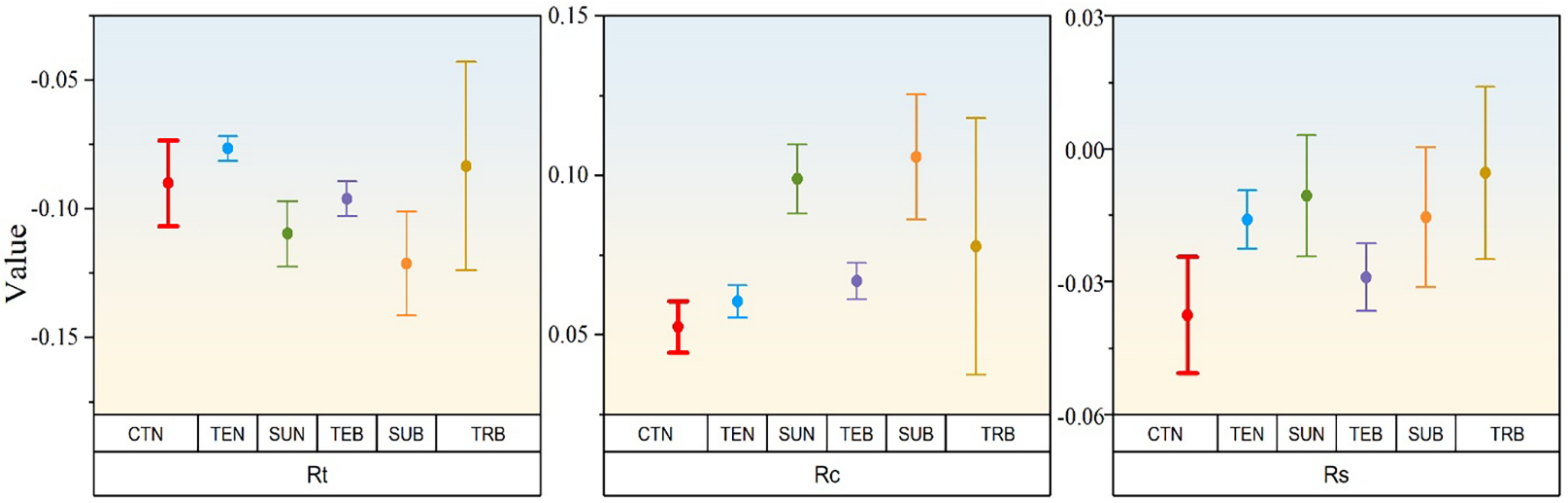
Value of resistance (Rt), recovery (Rc), and resilience (Rs) for different forest types during drought (according to Formulas (5), (6), (7)).

It shows the resistance, recovery, and resilience of broadleaf and coniferous forests in different temperature zones when the events of drought occur (Figure 6). The subtropical coniferous forests and broadleaf forests have relatively low resistance, and their recovery and resilience are relatively high. Cold‐temperate coniferous forests have a more pronounced weakness in resilience. Tropical broadleaf forests have relatively low resilience.

### 3.3 Assessment of complete vegetation restoration under high‐intensity drought events

**FIGURE 7:**
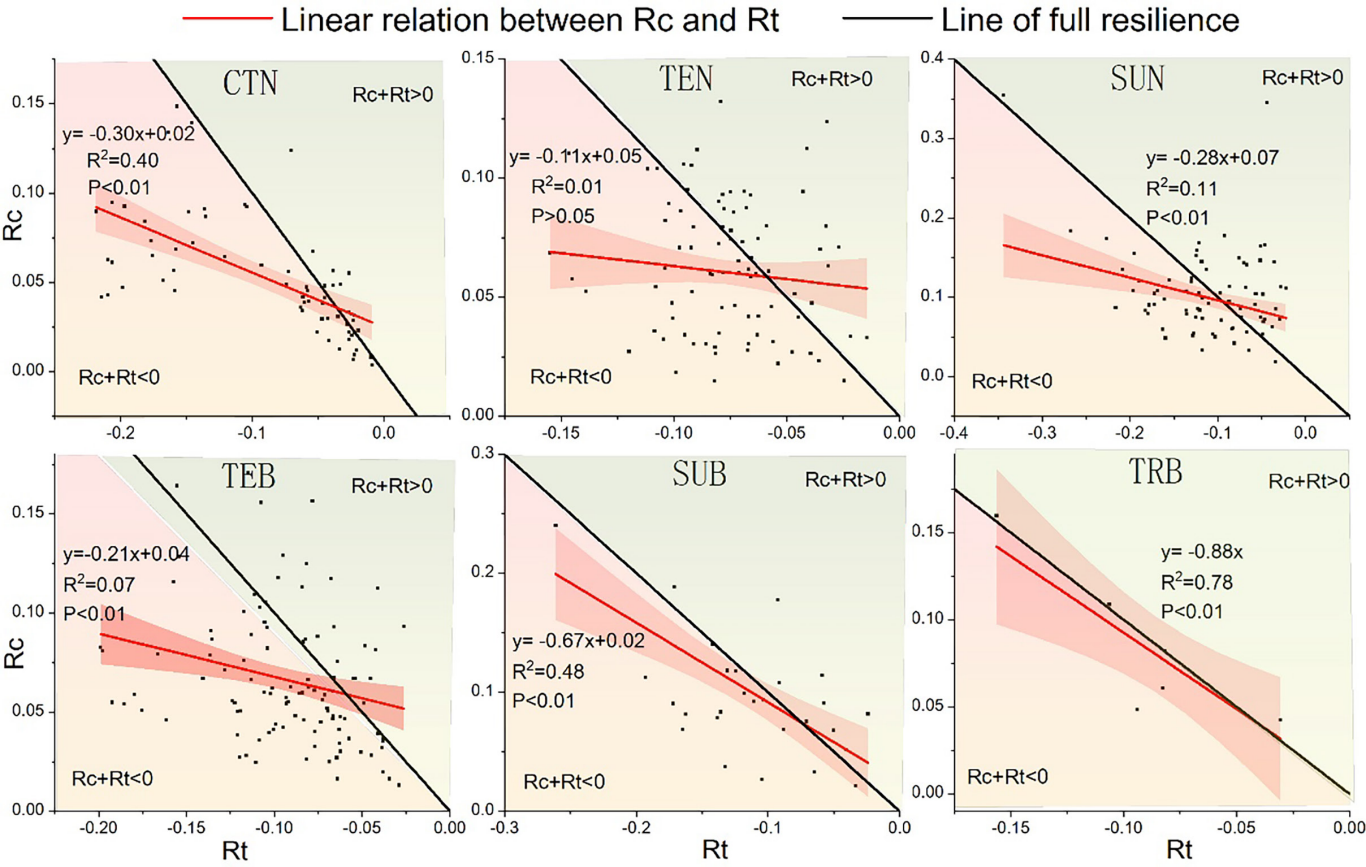
Comparing the ability of different forest types to cope with drought, red line represents the linear fitting relationship of resistance (Rt) and recovery (Rc), and black line represents full resilience at any given value of resistance.

It shows the linear fitting relationship of Rc and Rt for broadleaf and coniferous forests in different temperature zones when a high‐intensity drought event occurs (Figure 7). The *p*‐value of the fitting results is <.01 except for temperate coniferous forests. We can find that subtropical broadleaf forests and tropical broadleaf forests are the closest to the line of full resilience, while the fitting results of the other species are far apart.

## 4 DISCUSSION

### 4.1 Comparison of the effects of drought on vegetation in different temperature zones

Drought can have long‐term effects on internal factors from species to ecosystem scales. The legacy size and duration of these internal factors can be influenced by a series of external factors, including drought duration, intensity, and frequency (Müller & Bahn, 2022). In recent years, it has been increasingly recognized (Anderegg et al., 2020; Canarini et al., 2021), but our understanding of drought genetics and potential processes is still limited to specific aspects of plant and ecosystem functions such as radial tree growth (Galiano et al., 2011; Gazol et al., 2020). In this article, we attempted to explore the lagged effect of drought on NDVI, established a DLNM model, and estimated parameters based on cross basis coefficients. The results of the study found that the inhibitory effect of high intensity drought on vegetation NDVI showed an overall gradient along the drought intensity (Figure 3). The main sensitive lag time was the first 3 months, with the strongest lagged effect occurring in the month when the drought occurred.

Previous studies on quantifying the lagged effect of drought usually used correlation coefficients to calculate the vegetation index and SPEI on a month‐by‐month basis, selecting the maximum correlation coefficient (*p* < .05), and considering the corresponding lagged month as the lagged effect magnitude and time scale for the pixel (Wei et al., 2022; Xu et al., 2021). Due to the potential for covariance of exposures on neighboring dates, the accuracy of the estimation of lagged effects may be limited. In order to obtain higher accuracy in the estimation lagged effect curves, the DLNM model imposes a number of constraints, including the assumption of a constant effect over the lag interval or the use of continuous functions such as polynomials or spline to describe smooth curves (Gasparrini et al., 2010). We use the DLNM model to obtain the maximum drought impact and lag time of each pixel. After integration analysis, the results of different tree species are consistent, that is, the lagged effect of drought gradually decreases over time. It cannot be ruled out that drought has a significant lagged effect on some individuals. At present, there is a lack of results on the lagged effect curve of drought intensity over time, and it is still needed to make further researches.

### 4.2 Line of full resilience of coniferous and broad‐leaved forests

Generally speaking, areas with high frequency of drought occurrence have strong adaptability and ability to cope with water deficit (Zhang et al., 2023). The frequency of drought occurrence is relatively high in cold temperate coniferous forests, temperate coniferous forests, and temperate broad‐leaved forests, which have higher resistance to drought and weaker recovery (Figures 5 and 6). High resistance could result in low recovery or vice‐versa, but resilience would be the same in both cases (Lloret et al., 2011). The trade‐off between resistance and recovery has been confirmed in previous studies (Galiano et al., 2011). These plant species can adjust their substantial xylem characteristics related to hydraulic conductivity to enable them to cope with general drought events (Anderegg & Hillerislambers, 2016). However, it is still unknown whether its regulatory threshold exceeds its capacity range with climate change (Zhang et al., 2023).

To this end, we further analyzed the response of each tree species to drought events with a duration of drought and a recovery period of 3 months or more. Based on the full resilience framework, we fitted the relationship between the recovery and resistance of tree species in different temperature zones. We can find that subtropical broad‐leaved forests and tropical broad‐leaved forests are closest to the line of full resilience, and other species show significant deviations from the line of full resilience (Figure 7). At the same time, we also noticed that if the resistance value is low (below −0.1), the proportion of vegetation reaching the complete recovery line is very low when severe drought occurs, especially in the cold temperate and temperate zones (Figure 7a,b,d). Vegetation growth in the humid tropics is less affected by SPEI than in temperate and cold temperate regions (Huxman et al., 2004; Schuur, 2003). Although vegetation is generally highly resistant to SPEI in relatively arid temperate and cool‐temperate regions, when severe and extreme droughts occur, the rate of vegetation recovery is attenuated because arid woody species generally have generally slow growth rates (Bonet, 2004; Zhang et al., 2023). This finding strongly supports our results. In terms of species, we compared coniferous forests and broad‐leaved forests located in the same subtropical zone (temperate fitting results were not significant), indicating that subtropical broad‐leaved forests are closer to the complete recovery line. Broad‐leaved forests have a stronger ability to fully recover in the face of severe drought, which is consistent with previous conclusions (Schwarz et al., 2020; Yao et al., 2022). The different strategies for resisting drought between broad‐leaved and coniferous forests may be the main reason for this phenomenon (Anderegg et al., 2018; Gazol et al., 2017).

### 4.3 Limitations

This study attempts to propose a relatively systematic method for assessing the impact of drought and forest ecosystem resilience to drought, based on the distributed lag nonlinear model (DLNM) and the framework of line of full resilience. However, the rough spatial and temporal resolution of vegetation cover index data also limits the accuracy of assessing the impact of drought (Li, Piao, et al., 2020; Li, Tong, et al., 2020). Insufficient resolution of SPEI and NDVI data, which may cover other non‐forest areas. Meanwhile, vegetation types may undergo changes over a long research period, leading to potential uncertainty. Considering this, introducing ground tree observation data into the system may be an effective attempt. In addition to improving spatial and temporal resolution, it is also worth noting the individual characteristics of trees obtained through ground observations such as tree age. An observation showed that young trees have stronger resistance to low growth and long‐term growth than old trees in the Rocky Mountains. However, their ability to recover to pre‐drought levels may not necessarily be stronger (Lloret et al., 2011). These refinements help us to more fully understand drought impacts, lagged effects, and forest ecosystem resilience to drought.

## 5 CONCLUSION

This article develops a quantitative evaluation method that systematically analyzes the impact of drought on vegetation, the lagged effect of drought, and ecosystem resilience to drought. The main sensitive lag time of vegetation in each temperature zone is the first 3 months, with the strongest lagged effect occurring in the month of drought. The frequency of drought occurrence is relatively high in cold temperate coniferous forests, temperate coniferous forests, and temperate broad‐leaved forests. In response to high‐intensity drought events, subtropical broad‐leaved forests and tropical broad‐leaved forests have shown the strongest coping ability, with cold temperate coniferous forests, temperate coniferous forests, subtropical coniferous forests, and temperate broad‐leaved forests experiencing significant negative impacts. This study quantitatively reveals the impact and lagged effect of drought on forest ecosystem, providing a basis for forest health assessment and restoration in different temperature zones.

## AUTHOR CONTRIBUTIONS

**Qingfeng Xu:** Conceptualization (equal); data curation (equal); project administration (equal). **Ruyue Yu:** Data curation (equal); investigation (equal); writing – review and editing (equal). **Lili Guo:** Funding acquisition (equal); supervision (equal); writing – review and editing (equal).

## CONFLICT OF INTEREST STATEMENT

The authors declare that they have no known competing financial interests or personal relationships that could have appeared to influence the work reported in this paper.

## Data Availability

Data used for model fitting are available at can be obtained from the website (https://figshare.com/articles/dataset/data/26826391).
